# SIX1 Downregulation Suppresses Self-renewal Capacity and THY1 Expression in Hepatocellular Carcinoma and SIX1 Dominate the Survival in Liver Cancer

**DOI:** 10.5152/tjg.2023.22293

**Published:** 2023-08-01

**Authors:** Pelin Balçık-Erçin, Arzu Aysan, Nazlı Salık, Esma Erciyas

**Affiliations:** 1 Department of Molecular Biology and Genetics, Gebze Technical University Faculty of Fundamental Sciences, Kocaeli, Turkey; 2 Department of Biology, Dokuz Eylul University Faculty of Medicine, İzmir, Turkey

**Keywords:** SIX1, hepatocellular carcinoma, drug response, CD90, survival

## Abstract

**Background/Aims:**

Sine oculis homeoprotein 1 exerts an essential role in embryonic development, and it was also identified to be reactivated in various types of mammalian cancer. The sine oculis homeoprotein 1 transcription factor was demonstrated to induce epithelial–mesenchymal transition, regulate crucial genes associated with cancer progression, and increase the oncogenic potential of cells. Therefore, the present study aimed to identify the role of sine oculis homeoprotein 1 in cancer.

**Materials and Methods:**

Sine oculis homeoprotein 1 gene expression was tested with real-time quantitative polymerase chain reaction (PCR) in different cancer types. Sine oculis homeoprotein 1 expression was suppressed by short hairpin RNA transduction in the SNU398 hepatocellular carcinoma cell line. The effects of sine oculis homeoprotein 1 on cell proliferation, drug resistance, and sphere formation were assessed in shSIX1 cells. Immunohistochemical and in silico analyses were performed to determine the prognostic role of sine oculis homeoprotein 1 expression.

**Results:**

The upregulated expression levels of sine oculis homeoprotein 1 were revealed to be correlated with the stage of the disease in breast, colon, and liver cancer, with liver cancer exhibiting the highest expression profile. Sine oculis homeoprotein 1 downregulation significantly affected cell proliferation and suppressed sorafenib resistance and sphere-forming ability. Furthermore, sine oculis homeoprotein 1 knockdown cells were identified to have decreased CD90 levels, essential for cancer stem cell properties. Finally, sine oculis homeoprotein 1 expression was a CD90-independent biomarker for the clinical prognosis of liver cancer.

**Conclusion:**

The results of this study showed that the knockdown of sine oculis homeoprotein 1 expression might help to prevent hepatocarcinogenesis by increasing drug sensitivity and controlling tumor sphere formation. Overall, these results indicated that sine oculis homeoprotein 1 expression might be useful as a diagnostic marker for patients with hepatocellular carcinoma.

**Main Points**

- Sine oculis homeoprotein 1 (SIX1) transcription factor upregulated various cancers, especially liver cancer.
- Suppression of SIX1 causes hepatocellular carcinoma (HCC) cells to be sensitive to sorafenib.
- CD90 level and self-renewal capacity changes in HCC cells depend on SIX1 expression.
- The expression of SIX1 is a prognostic marker for liver cancer independent of CD90 expression.

## Introduction

Cancer is a major health problem worldwide, demonstrating ever-increasing incidence rates.^1^ Lung, breast, colorectum, and liver cancers are the top 4 types of cancer responsible for the most cancer-related deaths.^2^ Tumor metastasis, the process by which primary cancer disseminates to a distant organ and develops into a metastatic lesion, is still the leading cause of cancer-related mortality.^3^ Epithelial–mesenchymal transition (EMT) is a fundamental mechanism of cell migration, which has been associated with poor survival and resistance to chemotherapy in numerous types of cancers.^4^ The SNAI, TWIST, and ZEB (ZEB1 and ZEB2) family of transcription factors are the most thoroughly studied mediators of EMT in human cancers; however, other studies have identified new transcription factors that induced EMT, such as sine oculis homeobox 1 (SIX1).^5^ The overexpression of SIX1 in cancer was first observed in 1998, and it has since been discovered to be associated with a poor prognosis in numerous types of cancer.^6^ SIX1 was reported to affect tumor progression through the regulation of proliferation, invasion, metastasis, genomic instability, and resistance to cell death.^6,7^ In addition, SIX1 was previously demonstrated to mediate paclitaxel resistance in breast cancer cells.^8^ Drug resistance, a high rate of self-renewal, and the ability to differentiate are intrinsic properties of cancer stem cells (CSC), and it has been suggested that EMT and CSC properties are mechanistically related to each other in cells.^9^ Interestingly, recent studies have indicated that SIX1 served an important role in regulating CSC characteristics in various cancer cells.^10^

The goal of this study was to determine the expression profile of SIX1 in various types of cancer, in which the highest expression levels were detected in liver cancer. Furthermore, the results revealed that the expression levels of SIX1 were downregulated, which subsequently affected the drug resistance, self-renewal capacity, and decreased the expression levels of the CD90 of HCC cells. In liver cancer, SIX1 expression was a prognostic factor for survival that is independent of CD90 expression.

## Materials and Methods

### Cell Lines and Culture Conditions

Hepatocellular carcinoma cell lines SNU398, SNU182, and SNU475 were cultured in RPMI1640 supplemented with 10% fetal bovine serum. Hepatocellular carcinoma cell lines HUH7, HEP3B, PLC/PRF/5, and liver cancer cell line HEPG2 were cultured in Dulbecco’s Modified Eagle Medium (DMEM) supplemented with 10% fetal bovine serum at 37°C in a humidified 5% CO_2_ atmosphere. The cell lines were obtained from Dr. Tamer Yagci, which were previously used by Yalim-Camci et al.^11^ All cell lines were routinely checked for mycoplasma contamination. The identities of the cell lines were validated by Short Tandem Repeat (STR) analysis.

### Antibodies and Plasmids

The following antibodies and plasmids were used: rabbit polyclonal anti-SIX1 antibody (NOVUS, Seattle, Wash, USA) and mouse monoclonal CALNEXIN, c-PARP, β-ACTIN and ALFA-TUBULIN antibodies (Santa Cruz Biotechnology, Dallas, Tex, USA), cell signaling.shRNA lentiviral particles targeting human SIX1 (TRCN0000468669, Sigma-Aldrich, St. Louis, Mo, USA), and a non-silencing control (pLKO.1, Addgene #8453), packaging plasmids pCMV-dR8.2 dvrp (Addgene #8455) and pCMV-VSV-G (Addgene #8454).

#### *Production of Lentiviral Particles and Transduction to SNU398 Cell, Generation of SIX1-shRNA*,* and Control Clones*

Lentiviral SIX1 shRNA or control (pLKO.1) plasmids were combined with packaging plasmids and transfection agent PEI (Polysciences, Eppelheim, Germany) in Optimem medium to produce lentiviral particles (Thermo Fisher Scientific, Rockford, Ill, USA). The HEK293T cells were transfected and incubated for 36-48 hours after virus particles were extracted from the supernatant. SNU398 cells transduced with SIX1-shRNA and control-shRNA lentiviral particles in the presence of 8 μg/mL polybrene (#TR-1003-G, Sigma-Aldrich). After 24 hours, 5 µg/mL puromycin (Thermo Fisher Scientific, #A1113802) was added to the cultures to select stable shRNA and control clones.

### Cytotoxicity Assay

The shSIX1-SNU398 and control-SNU398 cells were plated in 96-well plates at an initial density of 5 × 10^3^ cells, with 100 µL media per well. After 24 hours of incubation, cell proliferation data were measured by MTT [3-(4,5-dimethylthiazol-2-yl)-2,5-diphenyltetrazolium bromide] assay (Thermo Fischer Scientific #M6494) at 12, 24, 36, and 48 hours.

### Western Blotting

Cells were lysed in Sodium Dodecyl Sulfate (SDS) lysis buffer (10% glycerol, 2% SDS in 62.5 mM Tris–HCl), including Protease Inhibitor Cocktail (Roche). After the determination of protein concentration, an equal amount of protein from each lysate was resolved by sodium dodecyl sulfate-polyacrylamide gel electrophoresis and transferred to a polyvinylidene difluoride membrane (Millipore, #IPVH00010). Membranes were incubated with primary antibodies at 4°C overnight, then incubated with the secondary antibody for 2 hours at room temperature. Finally, these membranes were immersed in SuperSignal West Femto chemiluminescent reagent (Thermo, #34095), and the protein bands were visualized with the ChemiDocTMXRS system (Bio-Rad, Calif, USA).

### Gene Expression Analysis

Total RNA was isolated from shRNA-SNU398 and control-SNU398 with Nucleospin RNA plus isolation kit (Macharey-Nagel, #740984.250). Complementary DNA was synthesized using the High-Capacity cDNA Reverse Transcription Kit (Applied Biosystems™, #4368814) according to the manufacturer’s instructions. The real-time quantitative PCR (RT-qPCR) reaction was performed using the Maxima SYBR Green qPCR master mix (Thermo Fisher Scientific, #K0223). Ct values were normalized to GAPDH and calibrated with Ct of controls. Relative gene expression was calculated by the ΔCt method. Control-SNU398 and shSIX1-SNU398 cells were analyzed by RT-qPCR by using the primers. Sequences of primers for cDNA amplification of *ZEB2* were 5’-CAAGGAGCAGGTAATCGCAAGT-3’ and 5’-GGAACCAGAATGGGAGAAACG-3’, *SIX1* primers were 5’-AAAGGGAAGGAGAACAAGGATAG-3’ and 5’-AGCCTACATGATTACTGGGATTT-3’. *THY1* primers were 5’-GTCCTTTCTCCCCCAATCTC-3’ and 5’-ACGAAGGCTCTGGTCCACTA-3’ *KLF4* primers were 5’-TCTCAAGGCACACCTGCGAA-3’ and 5’-TAGTGCCTGGTCAGTTCATC-3’. The primers for the normalizator *GAPDH *were 5’-GGCTGAGAACGGGAAGCTTGTCAT-3’ and 5’-CAGCCTTCTCCATGGTGGTGAAGA-3’.

### Sphere Formation Assay

Cells were seeded into ultra-low attachment surface plates at a concentration of 1.0 × 10^3^ cells/mL (Corning Inc., Corning, NY, USA). These cells were cultured in DMEM (Gibco, Carlsbad, CA, USA) supplemented with 50 ng/mL EGF (R&D Systems, Minneapolis, MN, USA), 10 ng/mL bFGF (R&D Systems, Minneapolis, MN, USA), 25 μg/mL insulin (Gibco), and 2% B27 supplement (Gibco) at 37°C in a humidified 5% CO_2_ atmosphere for 7 days. After incubation, images of spheroids were acquired with a Leica DMi8 inverted microscope, and the diameter of the spheroids was determined.

### Measurement of Cell Surface CD90 Expression with Flow Cytometry

Cells were resuspended at a density of 3 × 10^5^ cells/mL in phosphate-buffered saline (PBS) and transferred into new tubes, which were used for the control group and CD90-treated groups. After that, 5 µL FCR (Miltenyi Biotech) and CD90 (Sony) antibodies were added to the tubes and incubated at room temperature and samples were analyzed using Accuri C6 (BD Biosciences, San Jose, CA, United States) flow cytometer.

### Analyses of SIX1, CD90 Expression in Human Cancer Samples

The expression of SIX1 was assessed by RT-qPCR in commercial TissueScan qPCR Cancer Survey cDNA arrays I (Origene, #CSRT101). The array consists of 72 tumors and 24 non-malignant tissue samples from 8 different primary organs (breast, colon, kidney, liver, ovary, thyroid, lung, and prostate) and also provides clinical information. The expression of SIX1 in cDNA arrays was calculated by using the ΔΔCt method (Log2).^12^

The same tumor types were analyzed in the Expression Project for Oncology (expO) datasets (GEO accession GSE2109). In this study, R software was used for data manipulation, calculation, and graphical display (https://www.r-project.org).

Immunohistochemistry was carried out with the Hepatocellular Carcinoma Tissue Array, HLiv-HCC150PG-01 (Biomax, Rockville, MD, USA). The SIX1 protein expression level was evaluated by integrating the percentage of positive tumor cells and the intensity of positive staining. Briefly, sections were scored as 0 (negative), 1 (bordering), 2 (weak), 3 (moderate), or 4 (strong), whereas the staining extent was scored according to the area percentages: 0 (0%), 1 (1%-25%), 2 (26%-50%), 3 (51%-75%), or 4 (76%-100%). The products of staining intensity and extent scores were the final staining scores (0-16). The groups with a score of 0 were excluded from the analyses and did not participate in the statistics.

The expression of SIX1 and CD90 markers expression was assessed by the cancer genome atlas database (TCGA). The survival was analyzed in TCGA-paired human liver cancer samples.

### Statistical Analysis

GraphPad Prism statistical software was used to analyze the significance of the data. Gene expression levels in cell lines and TissueScan cDNA array were represented as mean + standard deviation (SD). Paired Student’s t-test was used for statistical analyses of data from gene and protein expression experiments in cell lines. Unpaired Student’s t-test was used for statistical analyses of the tissue array. The differential transcription profile of expO datasets was analyzed with the Kruskal–Wallis test, followed by the post hoc Dunn’s test.^13^ Significant differences were denoted as follows: *P < .05, **P < .01, and ***P < .001.

## Results

### The SIX1 Expression Levels Are Upregulated in Various Types of Cancer

**Figure 1.**
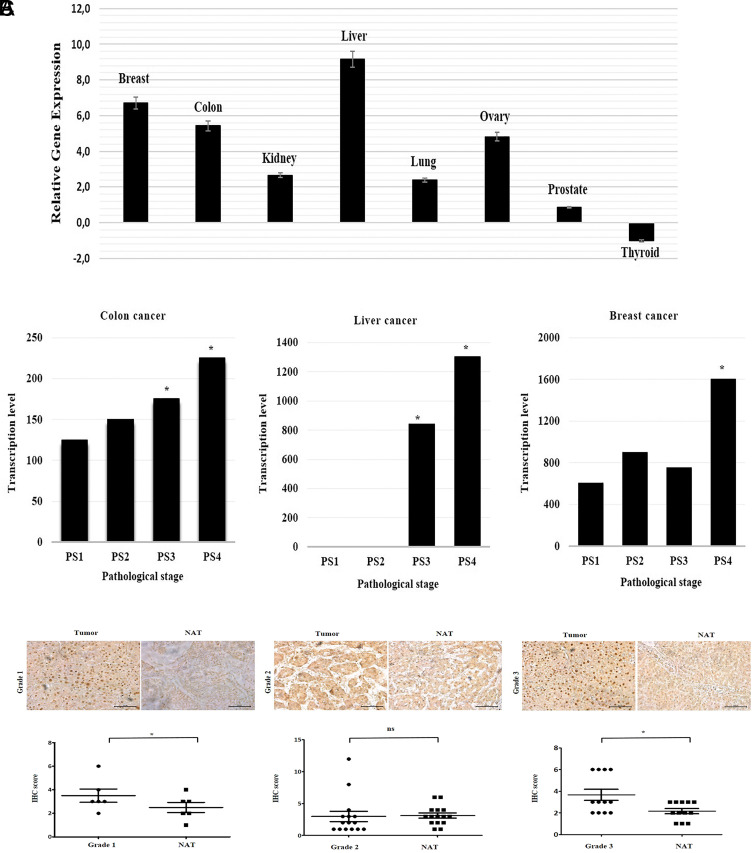
The SIX1 gene expression levels both in vivo and using bioinformatic analysis in different types of cancer.(A) Analysis of the expression levels of SIX1 in cancer cDNA arrays representing tissues from 3 non-malignant and 9 tumor samples from 8 different types of cancer. Compared with the control tissue, the expression levels of SIX1 in the liver, colon, breast, ovary, kidney, lung, and prostate tumor tissues were markedly upregulated, while they were slightly downregulated in thyroid cancer. mRNA expression levels were quantified using the 2^−ΔΔCq^ method. (B) mRNA transcription levels of SIX1 in colon, liver, and breast cancers depending on the pathological stages using an Expression Project for Oncology (expO) dataset. SIX1 expression was significantly upregulated in pathological stage 3 compared to pathological stage 1 in the liver and colon cancer, and also SIX1 high expression was detected in stage 4 compared to stage 1 in all 3 types of cancer (^*^ *P* < .05). (C) SIX1 expression levels in tumor tissues were compared with normal tissues in 3 different grades of hepatocellular carcinoma. SIX1 expression levels in grade 1 (n = 6) and grade 3 (n = 20) were significantly different in the tumor tissues compared with the normal tissues. In contrast, no significant differences were observed in grade 2 tissues (n = 12). NAT, non-associated tissue; SIX1, Sine oculis homeoprotein 1. ^*^ *P* < .05, unpaired Student’s *t*-test. Magnification, ×40; scale bar, 200 μm.

Previous studies reported that the upregulation of SIX1 expression levels was associated with a poor prognosis in breast, lung, pancreatic, cervical, colorectal, and ovarian cancers. Thus, the present study first analyzed the SIX1 expression pattern in liver, colon, breast, ovarian, kidney, lung, thyroid, and prostate cancers using cDNA arrays; the array represented tissues from 3 non-malignant samples and 9 tumor samples of 8 different cancer types. Compared with the control tissues, a marked upregulation in SIX1 expression levels was observed in the liver, colon, breast, ovary, kidney, lung, and prostate tumor tissues, while a slight downregulation was observed in thyroid cancer tissues (Figure 1A). Interestingly, liver, breast, and colon cancer tissues exhibiting high expression levels of SIX1 are very common, indicating that SIX1 upregulation may be associated with cancers with high mortality rates.^2^ Thus, the analysis of SIX1 expression levels was further investigated using expO datasets for tumor tissues of the liver, breast, and colon histopathological types, which were grouped according to the pathological stage. The statistical analyses of the colon, liver, and breast cancer transcriptional values were done by the Kruskal–Wallis test followed by post hoc Dunn’s test. Although SIX1 expression was significantly increased in pathological stages 3 and 4 compared to pathological stage 1 in liver and colon cancer samples, a significant increase was detected only in stage 4 compared to stage 1 in breast cancer (Figure 1B). Following these cDNA array results, the bioinformatics analysis also produced a similar SIX1 expression profile, with the highest expression levels detected in liver cancer.

The upregulation of SIX1 expression levels in liver cancer samples prompted the study into its expression levels with immunohistochemistry (IHC). The tissue array was treated with an anti-SIX1 antibody and analyzed according to the patient grade. Grade 1 and 3 IHC samples had significantly upregulated protein expression levels of SIX1 compared with the non-associated tissues (Figure 1C). The IHC results also validated the bioinformatics findings. Taken together, these data suggested that the upregulation of the SIX1 mRNA and protein expression levels may be an important indicator of the progression of liver cancer.

### The SIX1 Expression Levels Are Detected in Poorly Differentiated HCC Cell Lines, and SIX1 Knockdown Inhibits Cell Proliferation in the SNU398 Cell Line

Given the importance of the upregulation of SIX1 in numerous types of cancer, especially liver cancer, the role of the SIX1 transcription factor in HCC was subsequently investigated.

**Figure 2.**
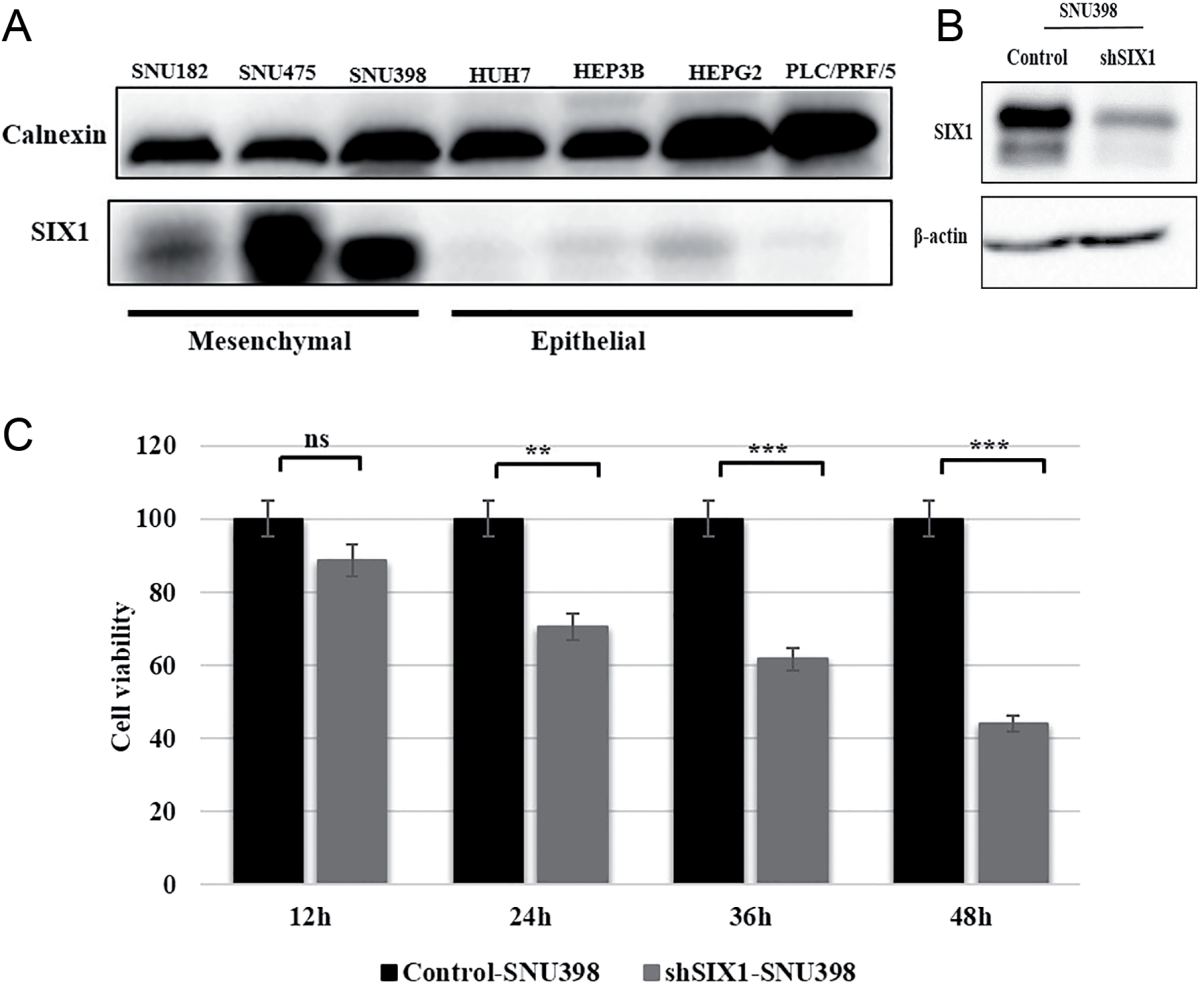
Expression levels of SIX1 in HCC cells and effects of SIX1 downregulation on proliferation in the SNU398 cell line. (A) Expression levels of SIX1 in different HCC cell lines with mesenchymal and epithelial characteristics. (B) The transfection efficiency of the knockdown of the SIX1 gene in the SNU398 cell line was validated using western blotting. β-Actin was used as the loading control. (C) Bars represent the proliferation of cells with and without SIX1 expression as determined by MTT assay. The differences between groups were analyzed using a Student’s *t*-test. HCC, hepatocellular carcinoma; MTT, [3-(4,5-dimethylthiazol-2-yl)-2,5-diphenyltetrazolium bromide]; ns, non-significant; SIX1, Sine oculis homeoprotein 1. ^***^ *P* < .001, ^***^ *P* < .0001.

The western blotting analysis identified that the poorly differentiated cell lines expressed the SIX1 protein, especially in SNU475, SNU398, and SNU182 cells (Figure 2A). According to our western blot result, the SIX1 protein may be present in SNU 475 in the hyperphosphorylated state, causing it to lose its ability to bind DNA.^14^ For this reason, SNU398 cells, which had the second-highest SIX1 expression levels, were selected for further analysis. SIX1-shRNA-SNU398 (shSIX1-SNU398) and plKo.1-shRNA-SNU398 (control-SNU398) stable clones were generated to determine the role of SIX1 in HCC cells (Figure 2B). To explore the effects of SIX1 knockdown on the proliferation capacity, we analyzed shSIX1-SNU398 and control-SNU398 HCC cell clone’s cell proliferation by MTT assay. Our results from cell viability assays were in line with previous studies,^15,16^ demonstrating that cellular proliferation is significantly inhibited in shSIX1-SNU398 at 24, 36, and 48 hours compared to control-SNU398 cells (Figure 2C)

### The SIX1 Knockdown Upregulates c-PARP Expression and the Apoptotic Cell Rate Following Sorafenib Treatment in HCC Cells

**Figure 3.**
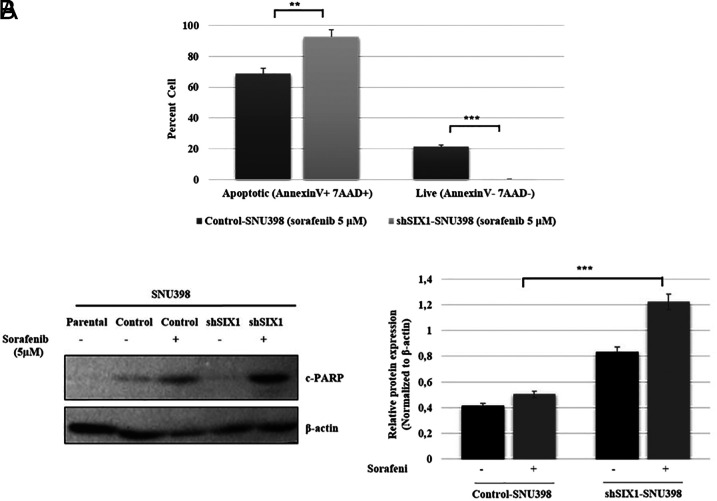
Effect of SIX1 knockdown on apoptosis and drug resistance in the presence of sorafenib. (A) Silencing of SIX1 promoted cell apoptosis. Apoptotic cells were analyzed using a BD Accuri C6 flow cytometer via Annexin PE/7-AAD staining. The percentage of apoptotic and living cells is shown. (B) Western blotting was used to investigate the expression levels of the c-PARP protein in SNU398 (parental), shSIX1-SNU398, and control-SNU398 cells. The c-PARP levels were increased in shSIX1-SNU398 cells compared to control-SNU398 cells with 5 µM sorafenib. β-Actin was used as the loading control. SIX1, SIX1, Sine oculis homeoprotein 1. ^**^ *P* < .001, ***P < .0001.

Epithelial–mesenchymal transition is a possible mechanistic basis for anti-cancer drug resistance.^17^ To the best of our knowledge, the SIX1-dependent effects on drug treatment have only been investigated in gastric and breast cancer cells.^8,18^ To further determine the effect of SIX1 in HCC, shSIX1 and control cell clones were treated with sorafenib, which is a standard chemotherapeutic drug for HCC. To understand the effect of SIX1 knockdown on drug resistance, flow cytometric analysis was performed, and it was noted that SIX1 knockdown cells were more sensitive to sorafenib treatment. The apoptotic cell number in shSIX1 and control cell clones was 93.9% and 66.7%, respectively, and the knockdown of SIX1 expression significantly increased the number of apoptotic cells (Figure 3A). Moreover, these findings were further confirmed by the subsequent protein changes. After exposure to sorafenib, the expression of cleaved PARP was increased in shSIX1-SNU398 cells compared to control-SNU398 and untreated cells (Figure 3B). These results indicated that SIX1 expression may exert a significant influence on the sorafenib drug response.

### Knockdown of SIX1 Expression Affects the Sphere-Forming Ability and CD90 Expression Levels

**Figure 4.**
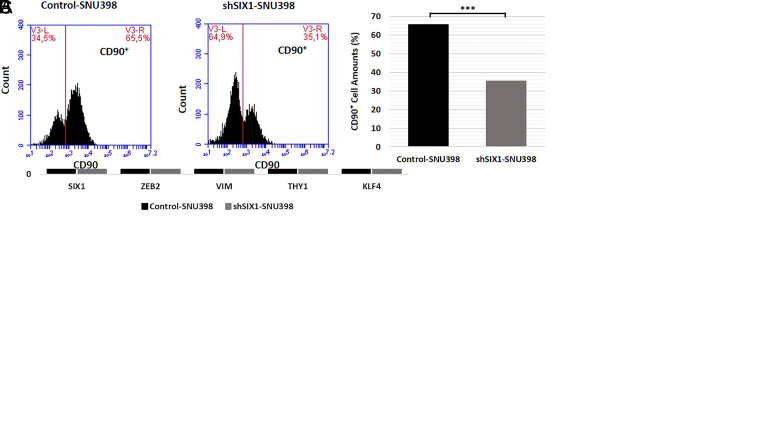
Determining the effect of SIX1 on sphere formation and self-renewal ability. (A) These cells were passaged 2 times to determine the effect of SIX1 on the self-renewal capacity following the sphere formation assay. It was subsequently observed that the shSIX1-SNU398 cells were unable to self-renew. (B) RT-qPCR was used to analyze the expression levels of *SIX1*, *ZEB2*, *VIM*, *KLF4*, and *THY1* genes in sphere-forming cells. (C) CD90 cell population in shSIX1-SNU398 and control-SNU398 cells was analyzed using flow cytometry. The CD90^+^ subpopulation was decreased in shSIX1-SNU398 cells compared with the control-SNU398 cells. The differences between groups were analyzed using a Student’s *t*-test. SIX1, SIX1, Sine oculis homeoprotein 1. ^**^ *P* < .01, ^***^ *P* < .0001. Magnification, ×5; scale bars, 200-μm.

To determine the role of SIX1 on the self-renewal capacity of HCC cell clones, a sphere formation assay was performed shSIX1-SNU398 sphere colony (>200 μm) and control-SNU398 colony (<65 μm) spheres are depicted. The control cells formed significantly increased numbers of spheres (>100 μm) compared with the SIX1 knockdown cells. Notably, following the second passage of sphere cells, shSIX1-SNU398 cell spheres did not retain the ability to self-renewal (Figure 4A).

To validate the morphological data, the mRNA expression levels of *SIX1*, *ZEB2*, *VIM, KLF4,* and *THY1* were analyzed in control and SIX1 knockdown sphere-forming cells. The results revealed that *ZEB2*, *VIM, KLF4,* and *THY1* gene expression levels were significantly downregulated in SIX1 knockdown sphere cells (Figure 4B).

A recent study demonstrated that the CSC marker CD90, a THY1 gene product, was important for the cell cycle, migration, invasion, and sphere-forming ability of HCC cells.^19^ The observed SIX1-dependent effects in the self-renewal capacity prompted the analysis of the CD90 cell population in shSIX1-SNU398 and control-SNU398 cells by flow cytometry; the knockdown of SIX1 significantly decreased the CD90^+^ subpopulation in shSIX1-SNU398 cell clones (35.1%) compared with the control-SNU398 cell clones (65.5%) (Figure 4C).

### The SIX1 Expression Negatively Correlates With the Overall Survival of Liver Cancer Patients and Survival Is Independent of CD90

Previous studies indicated that SIX1 overexpression was positively correlated with liver cancer prognosis and survival.^20,21^ A previous study showed that CD90 expressions significantly increase in liver tumor tissue compared to its paired normal tissue.^22^ The sphere analyses demonstrate that the SIX1 downregulation caused the inhibition of the CD90 protein level.

**Figure 5.**
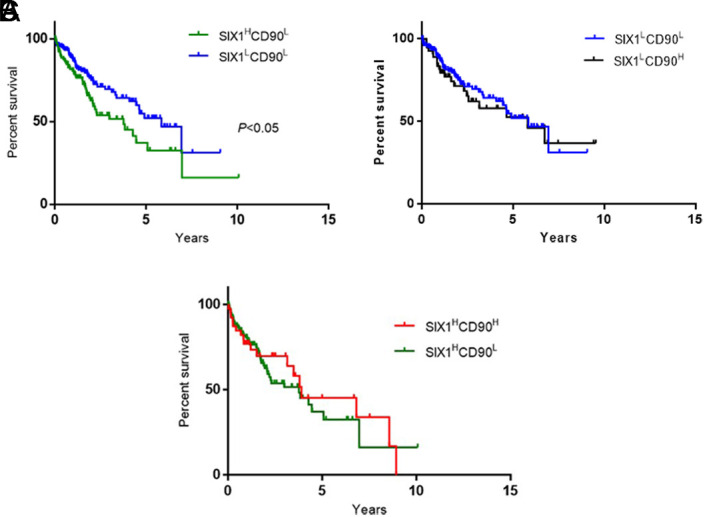
Kaplan–Meier survival curves for SIX1/CD90 conjoined expression in paired liver cancer samples: (A) Categorized by SIX1^H^CD90^L^ (n = 110) versus SIX1^L^CD90^L^ (n = 156) expression; (B) Categorized by SIX1^L^CD90^L^ (n = 156) versus SIX1^L^CD90^H^ (n = 58) expression; (C) Categorized by SIX1^H^CD90^H^ (n = 41) versus SIX1^H^CD90^L^ (n *=* 110). Survival was significantly poorest for patients with SIX1^H^CD90^L^ expression group compared to SIX1^L^CD90^L^ (*P* < .05). SIX1, SIX1, Sine oculis homeoprotein 1.

According to the conjoined expressions of SIX1/CD90, the patients were categorized into 4 groups: SIX1^H^CD90^H^, SIX1^H^CD90^L^, SIX1^L^CD90^H^, and SIX1^L^CD90^L^. The SIX1/CD90 survival rates were tested by the method of Kaplan–Meier. The results by pairwise comparisons showed that a statistically significant difference in survival rates existed between the SIX1^H^CD90^L^ patients and the SIX1^L^CD90^L^ group (Figure 5A) (*P* < .05). The conjoined expressions of SIX1^L^CD90^L^ vs SIX1^L^CD90^H^ (Figure 5B) and SIX1^H^CD90^H^ vs SIX1^H^CD90^L^ (Figure 5C) were not significantly associated with the patient’s survival rate.

## Discussion

Hepatocellular carcinoma is the most common type of liver cancer, demonstrating a high mortality rate.^23^ Several studies have reported that the expression levels of SIX1 were upregulated in various types of mammalian cancer and that the overexpression of SIX1 leads to increased motility of cancer cells, including in breast, colorectal, and hepatocellular carcinomas.^21^ In the present study, SIX1 expression levels were discovered to be upregulated in the different subtypes of cancer samples, especially in the liver, colon, and breast cancer tissues, compared with the non-malignant tissues in cDNA analyses. The present study further explored the expression of SIX1 in the *expO* dataset according to the patient stage in 3 types of cancer (liver, colon, and breast); interestingly, the expression levels of SIX1 were upregulated according to the patient stage. Thus, the upregulation of SIX1 expression levels appears to be important for liver, colon, and breast cancers, which are all placed in the top 5 cancers responsible for cancer-related mortalities.^2^ The findings of the present study are consistent with previous studies, and the reported SIX1 overexpression in our in silico and in vivo data suggested that SIX1 may be important for HCC progression.

Resistance to chemotherapy agents, limited available treatment options, and high mortality rates are associated with the incidence of HCC and prompted the present study to investigate the role of SIX1 in hepatocarcinogenesis in more detail. The results revealed that the SIX1 expression patterns were distinct in well and poorly differentiated HCC cell lines. Furthermore, SIX1 knockdown cells were more sensitive to sorafenib treatment; this result is compatible with previous studies. The SIX1 expression and drug resistance studies showed that SIX1 and drug responses are inversely related; SIX1 downregulation caused drug sensitivity, and SIX1 overexpression enhanced drug resistance in breast and ovarian cancer cells.^8,24^

Cancer stem cells share numerous characteristics with normal stem cells, including their differentiation and self-renewal abilities, and their drug-resistance properties.^25^ Previous studies have demonstrated that the SIX1 transcription factor affected the properties of CSCs in a variety of mammalian cancer cells; CSCs have several biological properties that distinguish them from other cancer cells, such as resistance to treatment, evasion from cell death, dormancy, and a sphere-forming ability.^26,27^ Compatible with the previous study,^27^ the spheres of SIX1 knockdown cells were significantly reduced in both the size and the number of spheres formed. Gene expression analysis of spheres formed from the SIX1 knockdown cell line showed that the mRNA expression levels of *ZEB2, VIM, KLF4*, and *THY1 *were markedly downregulated compared with the control cell spheres. Interestingly, a previous study demonstrated *THY1* expression was downregulated in ectopic SIX1 overexpress fibroblast tumors in RNA-Seq analysis.^28^ Zhang et al^19^ revealed that CD90, a gene product of *THY1* and a marker of HCC CSCs, and SIX1 suppression affected the CD90 positive cell count in the study. The CD90 is known to be important to mediate sphere-forming ability and is highly expressed in liver cancer tissues.^19,22^ We detected SIX1 overexpression more effected on liver cancer patient’s survival independent of CD90 expression profile.

The findings of this study revealed that SIX1 might be used as a biomarker for liver cancer progression. It may serve crucial roles in the drug resistance mechanism, cell renewal capacity, and survival rate in hepatocarcinogenesis. 

Future studies will be needed to test SIX1-dependent tumor stemness capacity in vivo models of HCC, and also additional studies will be required to assess the association between CD90 and SIX1 expression profiles in HCC cell colonies.

In conclusion, the current work extended our understanding of the functional roles of SIX1 in hepatocarcinogenesis and established the relevance of SIX1 as a biomarker and a therapeutic target.
